# Effect of a Sub-Chronic Oral Exposure of Broccoli (*Brassica oleracea* L. Var. *Italica*) By-Products Flour on the Physiological Parameters of FVB/N Mice: A Pilot Study

**DOI:** 10.3390/foods11010120

**Published:** 2022-01-04

**Authors:** Tânia Martins, Paula Alexandra Oliveira, Maria João Pires, Maria João Neuparth, Germano Lanzarin, Luís Félix, Carlos Venâncio, Maria de Lurdes Pinto, João Ferreira, Isabel Gaivão, Ana Isabel Barros, Eduardo Rosa, Luís Miguel Antunes

**Affiliations:** 1 Centre for the Research and Technology of Agro-Environmental and Biological Sciences (CITAB), University of Trás-os-Montes and Alto Douro (UTAD), 5000-801 Vila Real, Portugal; pamo@utad.pt (P.A.O.); joaomp@utad.pt (M.J.P.); glanzarin@utad.pt (G.L.); luis.felix@ibmc.up.pt (L.F.); cvenanci@utad.pt (C.V.); joaommf93@hotmail.com (J.F.); abarros@utad.pt (A.I.B.); erosa@utad.pt (E.R.); lantunes@utad.pt (L.M.A.); 2 Inov4Agro—Institute for Innovation, Capacity Building and Sustainability of Agri-Food Production, University of Trás-os-Montes and Alto Douro (UTAD), 5000-801 Vila Real, Portugal; 3 Department of Veterinary Sciences, School of Agrarian and Veterinary Sciences, University of Trás-os-Montes and Alto Douro (UTAD), 5000-801 Vila Real, Portugal; lpinto@utad.pt; 4 Research Centre in Physical Activity, Health and Leisure (CIAFEL), Faculty of Sports, University of Porto, 4200-450 Porto, Portugal; mneuparth@hotmail.com; 5 CEBIMED, Faculty of Health Sciences, Fernando Pessoa University, 4249-004 Porto, Portugal; 6 Instituto de Investigação e Inovação em Saúde (i3S), Laboratory Animal Science (LAS), Instituto de Biologia Molecular Celular (IBMC), Universidade do Porto (UP), 4200-135 Porto, Portugal; 7 Department of Animal Sciences, School of Agrarian and Veterinary Sciences, University of Trás-os-Montes and Alto Douro (UTAD), 5000-801 Vila Real, Portugal; 8 Animal and Veterinary Research Center (CECAV), University of Trás-os-Montes and Alto Douro (UTAD), 5000-801 Vila Real, Portugal; igaivao@utad.pt; 9 Department of Genetics and Biotechnology, School of Life and Environmental Sciences, University of Trás-os-Montes and Alto Douro (UTAD), 5000-801 Vila Real, Portugal

**Keywords:** broccoli flour, by-products, diet, FVB/N mice, glucosinolates, isothiocyanates

## Abstract

Brassica by-products are a source of natural bioactive molecules such as glucosinolates and isothiocyanates, with potential applications in the nutraceutical and functional food industries. However, the effects of oral sub-chronic exposure to broccoli by-product flour (BF) have not yet been evaluated. The objective of this pilot study was to analyse the effects of BF intake in the physiological parameters of FVB/N mice fed a 6.7% BF-supplemented diet for 21 days. Glucosinolates and their derivatives were also quantified in plasma and urine. BF supplementation significantly decreased (*p* < 0.05) the accumulation of perirenal adipose tissue. Furthermore, mice supplemented with BF showed significantly lower (*p* < 0.01) microhematocrit values than control animals, but no impact on the general genotoxicological status nor relevant toxic effects on the liver and kidney were observed. Concerning hepatic and renal antioxidant response, BF supplementation induced a significant increase (*p* < 0.05) in the liver glutathione S-transferase (GST) levels. In BF-supplemented mice, plasma analysis revealed the presence of the glucosinolates glucobrassicin and glucoerucin, and the isothiocyanates sulforaphane and indole-3-carbinol. Overall, these results show that daily intake of a high dose of BF during three weeks is safe, and enables the bioavailability of beneficial glucosinolates and isothiocyanates. These results allow further testing of the benefits of this BF in animal models of disease, knowing that exposure of up to 6.7% BF does not present relevant toxicity.

## 1. Introduction

Broccoli (*Brassica oleracea* L. var. *italica*), a member of the Brassicaceae family, is one of the most important vegetables, being grown all over the world [1]. In 2019, the European Union produced more than 2.3 million tonnes of broccoli and cauliflowers [2]. During broccoli harvesting, a large loss of material occurs. This loss encompasses not only the discarded inflorescences due to their small size, irregular shape, or damage, but also the leaves and stems, which represent about two-thirds of the plant [3]. These crop residues translate into economic losses for producers and environmental issues. Broccoli is a good source of glucosinolates and isothiocyanates, vitamins (A, C, K), folates, dietary fibres, phenolic compounds, and essential mineral nutrients [3,4,5,6]. The nutritional value and the presence of bioactive compounds with benefits for the prevention of obesity, carcinogenic, and cardiovascular diseases [7,8] contribute to the high popularity of this vegetable. In fact, the administration of extracts from broccoli inflorescences or stems have been shown to diminish the adipose tissue index and to increase glucose tolerance and insulin sensitivity in high fat diet (HFD)-fed rats [9]. Additionally, supplementation with a glucoraphanin-rich extract was able to decrease the accumulation of white fat tissue, hepatic steatosis, and oxidative stress, and improve glucose tolerance and insulin sensitivity in mice fed a HFD [10], thus showing the anti-obesity potential of this glucosinolate. In HFD-fed mice, supplementation with isothiocyanate sulforaphane (SFN) has also been shown to exert anti-obesogenic effects such as the attenuation of HFD-induced visceral adiposity and fat accumulation in the liver [11]. The discarded material during broccoli harvesting and processing is equally rich in these beneficial nutrients and phytochemicals [3,4,12]. Broccoli crop residues have been used for animal feed with positive outcomes. Egg quality [13], breast meat quality [14], and growth [15] were improved in laying hens and broilers with the incorporation of dried broccoli residues in diets. However, a higher concentration may compromise ileal and total tract nutrient digestibility [15]. Additionally, elevated consumption of glucosinolates have been linked to toxic effects in livestock [16]. In addition to animal feed, broccoli by-products can also be used for human consumption, since flours derived from these residues are suitable to be used to create functional foods [17,18,19,20].

Broccoli plants are rich in glucosinolates such as glucoraphanin and its reduced form glucoerucin, glucoiberin, and glucobrassicin, which are hydrolysed by the enzyme myrosinase after plant tissue damage or by the gut microbiota into their respective isothiocyanates, namely sulforaphane (SFN), erucin, iberin, and indol-3-carbinol (I3C), respectively [21]. These isothiocyanates are further metabolised into their derivatives, for instance, SFN is metabolised via the mercapturic acid pathway originating SFN-glutathione (SFN-GSH), SFN–cysteine (SFN–Cys), and SFN-*N*-acetylcysteine (SFN-NAC) [22], and I3C is converted to 3,3′-diindolylmethane (DIM) [23]. Nevertheless, a high consumption of broccoli or other *Brassicas* that are naturally rich in glucosinolates may cause some sort of toxicity. For instance, broccoli and other *Brassica* vegetables may interfere with thyroid hormone production, and people with hypothyroidism are recommended to avoid these vegetables [24,25]. In rats, the ingestion of total glucosinolates in a concentration range of 3.3–7.7 µmol/g of diet resulted in reduced intake and growth, increased thyroid weight, and led to thyroid morphology alterations [26,27,28,29]. These effects on thyroid function are due to the degradation products of glucosinolates [24,29]. In rats, glucosinolate levels below 120 μmol/kg of body weight per day do not trigger antinutritional or health risks, but at higher concentrations, adverse effects were observed for some glucosinolates [30]. 

Laboratory mice are the most widely used animal model in biomedical research, pharmacology, and immunology, with a view to its application in humans [31,32,33,34]. Thus, and considering the above-mentioned, in this study, we assessed the impact of sub-chronic dietary exposure to broccoli by-product flour composed by inflorescences, leaves, and stems in the physiological parameters of FVB/N mice, and evaluated the plasma and urine concentrations of glucosinolates and their derivatives.

## 2. Materials and Methods

### 2.1. Plant Material

Broccoli (*Brassica oleracea* L. var. *italica* cv. Naxus) plants were obtained from the producer Quinta do Celão, Unipessoal Lda, from a crop field located in Quinta das Abertas, Condeixa-a-Nova, Coimbra District, Portugal. Broccoli plants were harvested in October 2018. Plants were collected early in the morning and whole plants were carefully removed from the soil with their roots and soil, as much as possible, to extend the post-harvest life under transportation to the laboratory, and were carefully put in individual bags. At the laboratory, each plant was cut and divided into inflorescences, leaves, and stems (the roots were discarded) and kept at −80 °C. Then, intact plant material was freeze-dried and ground into flour in a food processor. Quantification of glucosinolates was carried out in each part of the broccoli plant individually (Supplementary Materials S1).

### 2.2. Diet Preparation

A commercial and standard diet for rodents (Mucedola 4RF21 Certificate, Milan, Italy) was used as a basis for the preparation of the modified diet containing broccoli by-product flour (BF). This experimental diet was formulated to contain 6.7% weight/weight (*w*/*w*) BF composed by 20% leaves, 35% inflorescence, and 45% stems. The dose of BF tested was established assuming that an adult person consumes around 150 g fresh broccoli per serving, which corresponds to around 19.05 g dry weight (dw) (according to our data, broccoli relative water content was 87.3%). The consumption of 19.05 g BF per person (60 kg) corresponds to a dose of 317.5 mg/kg. Applying a dose conversion formula between human (60 kg) and mouse (20 g) [35], the animal equivalent dose will be 3905.25 mg/kg in a mouse, corresponding to around 78 mg BF/mouse. Since this was a pilot toxicity study, only a 10 times higher dose of 780 mg BF/mouse was tested. Assuming that an adult person consumes on average three servings of broccoli per week, the intake of 780 mg BF three times a week is equivalent to the intake of 2340 mg BF/week/mouse, corresponding to an average daily intake of 334.23 mg BF/mouse. For a mouse with a 5 g average daily food intake, this corresponds to 6.7% of the daily feed. The commercial diet routinely used for mice colony maintenance was ground finely (1.2 mm) and mixed for 5 min with BF in a horizontal helix ribbon mixer (CPM Europe, C-300 model, Zaandam, The Netherlands), with 5% (*v*/*w*) water and dry pelleted at 35 °C (pellet size 4.8 mm in diameter). The control diet was prepared according to the same procedure but without including BF. All batches of feeds were then dried in a convection oven (48 h at 40 °C) and stored at 4 °C prior to utilisation.

### 2.3. Animals and Experimental Design

The animal experiments were approved by the Animal Welfare and Ethical Review Body of University of Trás-os-Montes and Alto Douro (UTAD) and by the national competent authority Direção-Geral de Alimentação e Veterinária (DGAV, Lisbon, Portugal; license no. 8776). FVB/N male mice (*n* = 10), aged 8-months, were obtained from a colony of UTAD’s animal facility. Mice were housed in open polycarbonate cages with corn cob bedding and environmental enrichment (cardboard rolls and paper) under controlled temperature (21 ± 2 °C), relative humidity (50 ± 10%), and a 12 h light (7 a.m. to 7 p.m.)/12 h dark (7 p.m. to 7 a.m.) cycle. Mice were assigned into two groups (*n* = 5/group) by simple randomisation using Excel. Mice were fasted for 12 h before the start of exposure to control (CTR) or 6.7% BF diets. All animals had ad libitum access to food and tap water. Animals were observed daily for any clinical signs of distress. Mice were weighed before the start of the experiment, after fasting and before euthanasia at the end of the study. The water and food intake were measured weekly. At the end of the experiment, after 21 days since the initiation of dietary exposure, mice were euthanised with an overdose of 150 mg/kg sodium pentobarbital (intraperitoneally; EUTHASOL^®^ 400 mg/mL, ESTEVE, Barcelona, Spain) followed by cardiac puncture and exsanguination. The urine was collected directly from the bladder. Vital organs (thymus, heart, lungs, spleen, kidneys, and liver), and perirenal and abdominal adipose tissues were collected and weighed.

### 2.4. Comet Assay

The alkaline comet assay was performed to quantify DNA strand breaks in mouse leucocytes [36,37]. The twelve-gel slide format (12 mini-gels/slide) was used as previously described [38]. Mouse fresh blood was collected by cardiac puncture and four mini-gels were analysed for each mouse. Briefly, slides were first coated with a layer of 1% normal melting point agarose (precoating). Upon deeply anaesthesia, 30 μL of blood were collected and mixed with 200 μL of PBS. Then, 60 μL of the blood–PBS mix was added to 200 μL of 1% low melting point agarose and 6 μL mini-gels were set on the precoated slides (12 mini-gels/slide). The slides were then left in a lysis solution (2.5 M NaCl, 0.1 M EDTA, 0.01 M Tris, 1% Triton X-100, pH 10) overnight at 4 °C. After lysis, two sets of slides were incubated in a humidified chamber (at 37 °C) for 30 min: one set incubated with formamidopyrimidine DNA glycosylase (FPG; which converts oxidised purines into additional DNA strand breaks) [37] diluted in enzyme buffer (0.04 M HEPES, 0.1 M KCl, 0.0005 M EDTA, 0.2 mg/mL bovine serum albumin, pH 8) and the other set incubated only with enzyme buffer. The next steps included the alkaline treatment—cells were immersed in an electrophoresis solution for 40 min (0.3 M NaOH, 0.001 M EDTA), and the electrophoresis—took place at 24 V (1 V/cm), 300 mA for 25 min. Upon nucleoid neutralisation, washing, and fixation, DAPI (1 µg/mL) was used for DNA staining. Each DAPI-stained mini-gel was observed in a fluorescence microscope (Olympus BX41 microscope; 400× magnification) and 100 comets were counted per mini-gel. Comets were visually scored as previously reported [39]. Each comet was ranked according to a standard scale (five classes ordered by tail intensity and length), from 0 (no tail; undamaged) to 4 (nearly all DNA in tail; severe damage). The final score was expressed as genetic damage index (GDI), ranging from 0 to 400 arbitrary units, calculated according to the following formula:

(1) $$\mathrm{GDI}=\left[\left(f_{i}\;\mathrm{class}\;0\;\mathrm{comets}\right)\;\times \;0\right]+\left[\left(f_{i}\;\mathrm{class}\;1\;\mathrm{comets}\right)\;\times \;1\right]+\left[\left(f_{i}\;\mathrm{class}\;2\;\mathrm{comets}\right)\;\times \;2\right]+\left[\left(f_{i}\;\mathrm{class}\;3\;\mathrm{comets}\right)\;\times \;3\right]+\left[\left(f_{i}\;\mathrm{class}\;4\;\mathrm{comets}\right)\;\times \;4\right]$$

The coefficient of variation (CV) was calculated for each condition as previously suggested [40]. CVs were inferior to 10% for all conditions, which allowed using a GDI for each condition. Thus, 20 replicates (four mini-gels; five mice) were considered. Considering the set of slides incubated with FPG, GDI was calculated the same way as before (expressed as GDI_FPG_). Additionally, the subtraction of GDI to GDI_FPG_ resulted in net FPG-sensitive sites (NSS_FPG_), which corresponds to the strand breaks induced specifically by the FPG activity (oxidised purines).

### 2.5. Haematology

For the microhematocrit analysis, the blood was collected into heparinised capillary tubes, and after centrifugation (13,548× *g*, for 5 min, RT), the column of red blood cells was read with a ruler. For plasma biochemical analyses, the blood was collected into lithium-heparin tubes and centrifuged at 1400× *g*, 15 min, 4 °C. The plasma was then collected and stored at −20 °C. The plasma concentrations of glucose, cholesterol, total proteins, creatinine, aspartate aminotransferase (AST), and alanine aminotransferase (ALT) were determined by spectrophotometric methods using an autoanalyzer (Prestige 24i, Cormay PZ). 

### 2.6. Histology

Vital organs (thymus, heart, lungs, spleen, kidneys, and liver) were collected and fixed by immersion in 10% buffered formalin. After fixation, organs were sectioned and routinely processed for paraffin embedding. Tissue sections 3 μm thick were stained with hematoxylin and eosin (H&E) and observed under a light microscope for histological analysis. The incidence of hepatic changes was classified according to its type (hydropic or vacuolar changes) and distribution (focal or diffuse). The presence of multifocal lymphocytic infiltrates in the liver and kidney were also registered.

### 2.7. Liver and Kidney Oxidative Stress

Liver and kidney samples were homogenised in cold buffer solution (0.32 mM of sucrose, 20 mM of HEPES, 1 mM of MgCl_2_, and 0.5 mM of phenylmethylsulfonylfluoride (PMSF), pH 7.4). Homogenised samples were then centrifuged at 15,000× *g* for 20 min at 4 °C, and supernatants were collected, and the analyses were performed in duplicate using a PowerWave XS2 microplate scanning spectrophotometer (Bio-Tek Instruments, Winooski, VT, USA) or Varian Cary Eclipse (Varian, Palo Alto, CA, USA) Spectrofluorometer, equipped with a microplate reader. The probe 2′,7′-dichlorofluorescein diacetate (DCFH-DA) was used to determine the generation of reactive oxygen species (ROS) with excitation at 485 nm and emission at 530 nm, as previously described [41]. The activity of total superoxide dismutase (SOD) was assessed according to the method described by Durak et al. [42] at 560 nm. The catalase (CAT) activity was estimated at 240 nm by a method previously described [42]. The activity of gluthathione S-transferase (GST) was assessed at 340 nm through the reaction of the thiol group of glutathione with 1-chloro-2,4-dinitrobenzene (CDNB). A molar extinction coefficient of 9.60 mM^−1^ cm^−1^ was used. The levels of reduce glutathione (GSH) and oxidised glutathione (GSSG) were determined by derivatisation with *ortho-phthalaldehyde* at 320 nm wavelength and 420 nm [43]. The oxidative-stress index (OSI) was determined by the ratio between GSH and GSSG. Malondialdehyde (MDA), an indicator of lipid peroxidation (LPO), was determined by the thiobarbituric acid (TBA)-based method [44] at 530 nm. Lactate dehydrogenase (LDH) activity was determined at 340 nm [45], and a molar extinction coefficient of 6.22 mM^−1^ cm^−1^ was used. 

### 2.8. Quantification of Glucosinolates, Isothiocyanates, and Their Metabolites

Pre-processing method for the analysis of glucosinolates in urine and plasma. Lyophilised urine and plasma samples were suspended in 0.2 mL ammonium acetate 13 Mm pH 7/0.1% formic acid in acetonitrile (50:50, *v*/*v*) by vortex and sonication for 10 min. Afterward, the samples were shacked using a vortex stirrer and centrifuged (10,000 rpm, 5 min, 4 °C). The supernatants were collected and filtered through a 0.22 μm Millex-HV13 membrane (Millipore Corp., Bedford, MA, USA).

### 2.9. UHPLC-ESI-QqQ-MS/MS Analysis

The identification and quantification of glucosinolates was conducted using ultra high-pressure liquid chromatography coupled to electrospray ionisation and a 6460 tandem mass spectrometer with triple quadrupole technology (UHPLC/MS/MS, Agilent Technologies, Waldron, Germany). The chromatographic separation was achieved by using a ZORBAX Eclipse Plus C18 (2.1 × 50 mm, 1.8 µm) (Agilent Technologies, Waldron, Germany) through a chromatographic gradient developed by applying different percentages of the solvents (A) 13 mM ammonium acetate pH 7 and (B) acetonitrile/formic acid (99.99:0.01, *v*/*v*), according to the multipurpose method optimised by Domínguez-Perles et al. [46], which separates intact glucosinolates, isothiocyanates, and their metabolites (Supplementary Table S1). The intact glucosinolates and isothiocyanates were identified following their *MS2* fragmentation pattern [mass/charge (*m*/*z*) ratio] by applying positive or negative ionisation mode depending on the compound considered at the optima ESI conditions for the maximal detection of the analytes, and their retention time in comparison with authentic standards (Supplementary Table S2). The glucosinolate contents of the urine and plasma samples analysed were expressed as nanograms per mL (ng/mL).

### 2.10. Statistical Analysis

Data are expressed as mean ± standard error of mean (SEM). Relative weight gain values are expressed in percentage. GDI, GDI_FPG_, and NSS_FPG_ are expressed in arbitrary units (a.u.). Statistical analysis was carried out using GraphPad Prism 7 (GraphPad Software, Inc., San Diego, CA, USA) software. To access if data followed a Gaussian distribution, data were analysed for normality by using the Shapiro–Wilk normality test. The *Chi*-square test was used for histologic lesion analysis. For the glucosinolate contents in broccoli plant parts, one-way ANOVA was performed followed by the Tukey’s multiple comparison test. For the rest of the analysis, the unpaired Student’s *t*-test was used. Differences were considered significant when *p* < 0.05. 

## 3. Results

### 3.1. General Findings

During the experimental trial, the animals were observed daily to assess their clinical condition. No changes were observed in the general appearance of the animals throughout the protocol.

Mice were fed a normal chow (CTR) or a diet with 6.7% BF, and were sacrificed at 21 days after starting the diet. Regarding food intake, both CTR (21.35 ± 0.93 g) and BF (20.40 ± 1.53 g) groups had similar average daily food intake values during the experimental protocol, without statistically significant differences. Additionally, both CTR (19.84 ± 0.85 mL) and BF (21.01 ± 1.22 mL) groups registered similar average daily water intake values without significant differences.

Concerning mean body weight (Supplementary Figure S1), there was a notorious significant initial lower body weight for the BF (30.36 ± 0.52 g; *p* < 0.001) group compared to CTR (37.90 ± 1.18 g). This difference remained until the end of the study, and was statistically significant between the BF (31.66 ± 0.60 g; *p* < 0.001) and CTR (37.35 ± 0.93 g) groups. Mice were fasted overnight before exposure to the new diet, and the weight loss after the fasting period was proportionally similar for both the CTR (34.15 ± 1.03 g) and BF (26.86 ± 0.57 g) groups and significantly different (*p* < 0.001). Mice regained their initial body weight after one week, which was maintained until the end of the study. The variation in body weight throughout the experiment, except after fasting, was very small for both groups, and the relative weight gain was not statistically different between the CTR (−1.5 ± 1.8%) and BF (4.0 ± 1.6%) groups.

### 3.2. Comet Assay

Genetic damage index (GDI) with and without formamidopyrimidine DNA glycosylase (FPG; GDI_FPG_) and net FPG-sensitive sites (NSS_FPG_) were measured on mice leucocytes (Supplementary Figure S2). The GDI values were not significantly different between exposure to BF (169.80 ± 2.20 a.u.) and CTR (167.35 ± 2.30 a.u.). The incubation with FPG resulted in the expression of GDI_FPG_, composed of GDI and NSS_FPG_ (specific damage induced by FPG), which detects potential oxidative DNA damage to leukocytes. Therefore, as expected, GDI_FPG_ values were increased relative to GDI values. However, non-significant differences between the CTR (191.60 ± 2.55 a.u.) and BF (197.70 ± 2.58 a.u.) groups were also found regarding GDI_FPG_. Given the damage increments for GDI_FPG_ values relative to GDI values, which correspond to the NSS_FPG_ values, a significant increase was shown for the BF (27.90 ± 0.88 a.u.; *p* < 0.01) group when comparing NSS_FPG_ values with the CTR (24.25 ± 0.89 a.u.) group.

### 3.3. Haematology

The microhematocrit analysis (Table 1) revealed a significant decrease (*p* < 0.01) in the BF group values compared to CTR. Regarding plasma biochemical parameters (Table 1), the differences in the plasma levels of glucose, cholesterol, total proteins, creatinine, ALT, and AST were not statistically significant between the CTR and BF groups. 

### 3.4. Organs and Adipose Tissue Weight

Table 2 shows the relative weight of vital organs, demonstrating a significant increase in the spleen (*p* < 0.05) and a significant decrease in the right kidney (*p* < 0.05) relative weights of the BF group compared to CTR. The BF group showed a reduction in the relative weight of adipose tissues, however, only the difference in the perirenal adipose tissue (*p* < 0.05) was statistically significant from CTR. 

### 3.5. Liver and Kidney Histology

No relevant liver and kidney histologic lesions were observed for the BF group compared to CTR (Supplementary Figure S3). Regarding hepatic changes, only focal hydropic degeneration (zone 3) in five mice (100%) of the CTR group and three mice (60%) in the BF group was observed. Multifocal lymphocytic infiltrate was observed in two mice (40%) of the BF group without statistical significance between groups. Regarding renal lesions, the multifocal lymphocytic infiltrate was identified in four mice (80%) in the CTR group and in one mouse (20%) in the BF group, also without significant differences between groups.

### 3.6. Liver and Kidney Oxidative Stress

Regarding the oxidative stress parameters in the liver and in the kidney (Table 3), in general, no significant differences were observed between the CTR and BF groups for each time point except for a significant increase in GST activity in the liver of the BF group compared to CTR. 

### 3.7. Glucosinolates, Isothiocyanates, and Metabolic Derivatives in Urine and Plasma

Table 4 summarises the concentration (ng/mL) of the individual intact glucosinolates and isothiocyanates and their metabolic derivatives identified in the plasma and urine samples. With respect to the concentration of glucosinolates and isothiocyanates in blood plasma in mice supplemented with BF for 21 days, four out of the twelve compounds analysed (four glucosinolates and eight isothiocyanates and metabolic derivatives) were found, namely glucoerucin, glucobrassicin, SFN, and I3C (Table 4). These compounds, SFN and I3C, undergo further phase II metabolisation or dimerisation before being excreted by urine, where according to the results presented (Table 4) would give rise to SFN-GSH, SFN-NAC, or DIM, depending on the isothiocyanate considered. The compounds SFN-NAC and DIM were found in all urine samples of the BF-supplemented group; however, the concentration in the CTR samples indicates that in these urines, the amount quantified was due to the background of the analytical technique. Besides SFN-NAC and DIM, the urine samples of the BF-supplemented group also exhibited the presence of non-esterified SFN, SFN-GSH, and I3C (Table 4). 

## 4. Discussion and Conclusions

Lately, the scientific community is paying attention to the potential use of plant and fruit by-products as a source of beneficial bioactive compounds [47]. Some studies have drawn attention to the usefulness of broccoli by-products in the production of functional foods enriched with bioactive compounds for human consumption [17,18,19,20,48]. However, despite the health benefits of broccoli, its high consumption may also present some health risks due to their high content in glucosinolates [24,26,27,28,29,30]. In this study, we evaluated the potential sub-chronic toxic effects of 6.7% BF supplementation for 21 days in healthy adult mice. During the present study, no physiological or behavioural changes were observed, demonstrating that BF supplementation is well tolerated by mice. The results of the study also showed that BF supplementation did not interfere with food or water intake and neither with the relative weight gain of mice. These results agree with Chen et al.’s [49] studies with 10% broccoli supplementation for 31 weeks, which showed no influence on the intake and body weight of mice. Ideally, the animals should have similar body weight at the beginning of the experiment, however, our experimental groups showed a difference of around 7 g, this disparity is probably a result of the randomisation method used [50]. Additionally, mice maintained their weight during the study, since at eight months of age, mice are expected not to gain more weight naturally. 

Regarding the genotoxicological evaluation by the comet assay, the ingestion of BF for 21 days did not have an impact on the overall genotoxicological status of mice, with and without FPG incubation (GDI_FPG_ and GDI, respectively). Nevertheless, genotoxic effects were demonstrated when isolating DNA oxidative damage recognised by FPG (NSS_FPG_). Accordingly, as shown in previous studies with other food products, a minor increase in oxidative damage may occur to activate the antioxidant system, leading to better prepared cells against severe genotoxicological challenges [51,52]. Previous studies have demonstrated the induction of genotoxic effects by broccoli (including raw material, extracts, and isolated compounds) in different in vitro and in vivo systems using a battery of genotoxicological assays [24,53]. The genotoxic activity of broccoli is also due to the degradation products of glucosinolates [24]. In contrast, broccoli showed the absence of genotoxicity, and even antigenotoxicity, in several other in vitro and in vivo studies [24,53]. Thus, further studies should be performed to clarify the genotoxicological impact of broccoli.

The microhematocrit analysis revealed a significant decrease in red blood cells of around 3% in the BF-supplemented group compared to CTR. However, the values presented for both experimental groups were within the physiological parameters, since hematocrit values are highly variable and may range from 35% to 52% in healthy mice [54]. In terms of biological significance, this suggests that it is likely that this reduction in the number of red blood cells may not reflect a real anaemia induced by BF supplementation. However, adverse effects in blood parameters induced by glucosinolates present in BF may not be ruled out as basal microhematocrit values were not determined at the beginning of the study. Concerning the plasma biochemical parameters analysed, BF supplementation did not induce changes in glucose or cholesterol plasma levels. There were also no significant changes concerning the levels of total proteins, creatinine, and the hepatic transaminases ALT and AST, suggesting that dietary supplementation with BF does not induce kidney or liver toxicity at the biochemical level. In agreement, the histological analysis did not reveal statistical differences between the CTR and the group supplemented with BF. Additionally, the relative weights of the left kidney and liver were not statistically different between the two groups. Although the relative weights of the right kidney and spleen were statistically different between groups, the increase seen in the BF-supplemented group was only very slight. In accordance with the results from biochemical and histological analysis, in general, BF supplementation did not induce renal and hepatic oxidative stress compared to CTR. However, an increase in liver GST activity was observed in animals supplemented with BF. This may be due to the fact that broccoli plants are rich in the glucosinolate glucoraphanin, the precursor of the isothiocyanate SFN [3,55], which is an inducer of liver GST [56]. 

Another finding from the present study is the lower relative weights of perirenal and abdominal adipose tissue in mice supplemented with BF compared to CTR, without alteration of food intake. However, only the difference for the perirenal adipose tissue was statistically significant. Although there was a body weight difference between both experimental groups observed from the beginning of the study, the calculation of the relative weight tries to remove the influence of this factor. Some in vivo and in vitro studies have demonstrated that glucoraphanin or SFN may exert anti-obesity effects [8]. For instance, a study in mice fed a HFD showed that ingestion of 0.1% SFN for six weeks attenuated HFD-induced increase in fat accumulation in the liver, and weight gain in mice, which was accompanied by a decrease in perirenal and epididymal adipose tissue weights and a reduced size in epididymal adipocytes [11]. Another work demonstrated that mice fed a HFD supplemented with 0.3% glucoraphanin for 14 weeks showed a reduction in weight gain, without altering food intake, and presented a decrease in adiposity and an increase in energy expenditure [10]. Similarly, Xu et al. [57] also showed that glucoraphanin (150 μmol/kg of body weight) decreased bodyweight, epidydimal, retroperitoneal, and liver weight in mice fed an HFD. In our study, the experiment was conducted on healthy animals not overweight, probably the reason why the effects were mild. Nevertheless, our finding may suggest that BF-supplementation can help with weight loss.

Glucoraphanin and its reduced forms glucoerucin, glucoiberin, and glucobrassicin, are some of the glucosinolates that are present in broccoli plants. These glucosinolates are hydrolysed by the enzyme myrosinase after plant tissue damage or by the gut microbiota into their respective isothiocyanates, which are further metabolised into their derivatives [21]. Glucoraphanin is hydrolysed to SFN, which is then conjugated with glutathione (GSH) to form SFN-GSH. This conjugate is subsequently metabolised via the mercapturic acid pathway to SFN–cysteine (SFN–Cys), culminating in the formation of the final metabolite SFN-*N*-acetylcysteine (SFN-NAC) [22]. SFN-NAC is the major metabolite of SFN appearing in urine, and is often used as a marker of bioavailability, although it is not the only metabolite present in urine, and intact glucosinolates may also be present [58,59,60,61,62]. Erucin is derived from the hydrolysis of glucoerucin, but it can also be formed through the reduction in SFN [22,60]. Glucoiberin, after glucoraphanin, is one of the major glucosinolates present in broccoli, and its hydrolysis gives rise to iberin [63]. Erucin and iberin are also further metabolised via the mercapturic acid pathway [58,60]. Glucobrassicin is the parent compound of I3C, which under acidic conditions is converted into polymeric products of which DIM is the main one and a final product [23]. Our data regarding the glucosinolate contents of broccoli inflorescences, stems, and leaves (Supplementary Table S3) show that the aliphatic glucosinolate (glucoiberin and glucoraphanin) contents were similar between inflorescences, stems, and leaves. For indolyl glucosinolates, the contents of 4-hydroxyglucobrassicin and 4-metoxyglucobrassicin were also similar, except for glucobrassicin and neoglucobrassicin, where the values were higher in the inflorescences and leaves compared to stems. These data demonstrate that both broccoli leaves and stems are by-products with high interest and potential for exploitation due to their high glucosinolate contents. In our study, we also proceeded to the quantification of glucosinolates and isothiocyanates in plasma and urine. Our results demonstrated that mice supplemented with BF showed circulating plasma levels of the intact glucosinolates glucoerucin and glucobrassicin, and of the isothiocyanates SFN and I3C, demonstrating the bioavailability of these compounds after daily ingestion of 6.7% BF for three weeks. In our study, besides glucoerucin, glucobrassicin, SFN, and I3C, the derivatives SFN-GSH, SFN-NAC and DIM, were also excreted in the urine, demonstrating that SFN and I3C underwent metabolisation. Glucoraphanin was under the detection limit in the plasma as well as in urine, probably due to its high rate of hydrolysis into SFN and conversion to its reduced form glucoerucin. In Budnowski et al.’s [60] study, glucoraphanin (172 mg/kg body weight) was administered intragastrically to germ free and human microbiota associated mice, without and with myrosinase application. In general, intact glucoraphanin was found in urine at higher levels than glucoerucin, SFN and SFN-NAC [60], which is in contrast with our results. Additionally, SFN and erucin were mainly excreted as urinary N-acetyl-L-cysteine conjugates, but glutathione or cysteine conjugates were not detected [60]. In our study, although glucoerucin was present in plasma and urine, erucin was under the detection limit, and the mercapturic acid conjugates of erucin were not determined to confirm its metabolisation. These differences may be due to differences in the compounds and doses administered, time of exposure, administration route as well as animals used, since high interindividual differences in the metabolism of the glucosinolates were also observed [60]. In our study, SFN-GSH was present in urine, but was under the detection limit in the plasma. In contrast, a previous study showed that after broccoli sprout (2.5 mg/g body weight) administration by gavage to mice, the absorption of SFN and its conjugation to glutathione to form SFN-GSH was fast, with both peaking in plasma at 0.5 h [64]. This conjugate was the major means of transport of SFN throughout the body, reaching higher concentrations than SFN in plasma, lungs, heart, liver, and muscle, except in the kidney, and was under the detection limit in mammary fatpad [64]. Once again, these variations between studies may be due to differences in the compounds and doses administered, time of exposure as well as administration route. Nevertheless, overall, our results demonstrate that BF ingestion leads to the bioavailability of glucosinolates and their derivatives.

In conclusion, our results show that, in general, the daily intake of a high dose of broccoli by-product flour during three weeks is safe and leads to the bioavailability of glucosinolates, having no negative impacts in the mouse’s health. However, being a pilot study, this work has some limitations such as the low number of animals, the testing of a single dose, and a relatively short exposure time. Nevertheless, with these results, further studies can be carried out, namely to evaluate the beneficial effects of this broccoli by-product flour in animal models of disease such as obesity, knowing that sub-chronic oral exposure of a dose up to 6.7% BF does not present toxicity in the evaluated parameters.

## Figures and Tables

**Table 1:** Microhematocrit and plasma biochemical parameters.

	CTR	6.7% BF
Microhematocrit (%)	43.21 ± 0.65	40.04 ± 0.55 **
Glucose (mg/dL)	335.60 ± 26.78	329.50 ± 42.27
Cholesterol (mg/dL)	157.20 ± 9.74	169.70 ± 40.68
Total Proteins (g/L)	45.06 ± 1.94	41.32 ± 4.16
Creatinine (mg/dL)	0.20 ± 0.06	0.36 ± 0.15
AST (U/L)	115.30 ± 23.39	97.66 ± 26.64
ALT (U/L)	29.00 ± 3.31	31.52 ± 5.89

ALT, alanina aminotransferase; AST, aspartato aminotransferase. Mice were fed a normal chow (CTR) or a diet with 6.7% (*w*/*w*) broccoli by-product flour (BF) and sacrificed after 21 days. Data are expressed as mean ± SEM (*n* = 5). ** *p* < 0.01, significantly different from the CTR group for the same parameter, according to the unpaired Student’s *t*-test.

**Table 2:** Relative weights of organs, perirenal, and abdominal adipose tissues (mg/g of body weight).

Organs	CTR	6.7% BF
Thymus	1.13 ± 0.16	0.90 ± 0.26
Heart	4.45 ± 0.16	4.75 ± 0.44
Spleen	3.00 ± 0.14	3.60 ± 0.19 *
Lungs	6.83 ± 0.39	6.05 ± 0.48
Right Kidney	8.60 ± 0.21	7.78 ± 0.22 *
Left Kidney	7.61 ± 0.24	7.32 ± 0.27
Liver	45.75 ± 3.23	49.81 ± 1.03
Perirenal adipose tissue	5.82 ± 0.69	3.29 ± 0.62 *
Abdominal adipose tissue	17.16 ± 2.95	12.89 ± 2.57

Mice were fed a normal chow (CTR) or a diet with 6.7% (*w*/*w*) broccoli by-product flour (BF) and sacrificed after 21 days. Data are expressed as mean ± SEM (*n* = 5). * *p* < 0.05, different from CTR for the same parameter, according to the unpaired Student’s *t*-test.

**Table 3:** Hepatic and renal oxidative stress parameters.

	Liver		Kidney	
Oxidative Stress Parameters	CTR	6.7% BF	CTR	6.7% BF
ROS(µmol DCF mg^−1^ protein)	674.3 ± 108.5	657.2 ± 132.3	595.9 ± 94.7	508.5 ± 69.5
SOD(U mg^−1^ of protein)	554.9 ± 106.8	550.1 ± 96.9	476.2 ± 63.6	511.1 ± 42.0
CAT(U mg^−1^ of protein)	608.1 ± 90.1	467.2 ± 153.3	181.7 ± 32.3	188.1 ± 35.4
GST(nmol CDNB per min^−1^ mg^−1^ of protein)	195.9 ± 26.7	379.5 ± 61.7 *	21.7 ± 2.1	23.9 ± 3.8
GSH(µmol GSH mg^−1^ of protein)	41.3 ± 5.2	51.0 ± 5.1	54.2 ± 9.0	44.9 ± 6.9
GSSG(µmol GSSG mg^−1^ of protein)	40.1 ± 4.2	49.7 ± 8.9	17.7 ± 6.2	24.2 ± 2.5
OSI	1.0 ± 0.1	1.1 ± 0.2	4.9 ± 1.4	1.9 ± 0.2
LPO(µmol MDA mg^−1^ of protein)	36.5 ± 3.8	40.1 ± 8.8	42.7 ± 6.6	36.9 ± 2.7
LDH(nmol NADH per min^−1^ mg^−1^ of protein)	18.7 ± 1.1	21.3 ± 4.1	5.0 ± 2.1	4.84 ± 1

ROS, Reactive oxygen species; SOD, Superoxide dismutase; CAT, Catalase; GST, Glutathione S-transferase; GSH, Reduce glutathione; GSSG, Oxidised glutathione; OSI, Oxidative stress index; LPO, lipid peroxidation; LDH, lactate dehydrogenase. Mice were fed a normal chow (CTR) or a diet with 6.7% (*w*/*w*) broccoli by-product flour (BF) and sacrificed after 21 days. Data are expressed as mean ± SEM (*n* = 5). * *p* < 0.05, when compared with the respective CTR group for the same parameter, according to the unpaired Student’s *t*-test.

**Table 4:** Plasma and urine concentrations of glucosinolates, isothiocyanates, and their metabolic derivatives.

	Plasma		Urine	
Compounds Detected (ng/mL)	CTR	6.7% BF	CTR	6.7% BF
Glucoraphanin	N.d.	N.d.	N.d.	N.d.
Glucoerucin	N.d.	46.6 ± 1.6	N.d.	995.6 ± 223.2
Glucoiberin	N.d.	N.d	N.d.	N.d.
Glucobrassicin	N.d.	159.9 ± 20.2	N.d.	2301.0 ± 452.8
SFN	N.d.	27.4 ± 1.6	N.d.	40.2 ± 9.2
Erucin	N.d.	N.d.	N.d.	N.d.
Iberin	N.d.	N.d.	N.d.	N.d.
I3C	N.d.	88.9 ± 6.8	N.d.	812.6 ± 118.8
SFN-GSH	N.d.	N.d.	N.d.	39.5 ± 7.5
SFN-CYS	N.d.	N.d.	N.d.	N.d.
SFN-NAC	N.d.	N.d.	0.5 ± 0.2	75.9 ± 22.5
DIM	N.d.	N.d.	54.3 ± 22.1	1498.0 ± 197.4

SFN, Sulforaphane; I3C, indole-3-carbinol; SFN-GSH, Sulforaphane-glutathione; SFN-CYS, Sulforaphane-cysteine; SFN-NAC, Sulforaphane-*N*-acetyl-cysteine; DIM, 3,3′-Diindolylmethane; N.d., not detected. Mice were fed a normal chow (CTR) or a diet with 6.7% (*w*/*w*) broccoli by-product flour (BF; composed by 20% leaves, 35% inflorescence, and 45% stems) and sacrificed after 21 days. Data are expressed as mean ± SEM (*n* = 5).

## Supplementary Materials

The following supporting information can be downloaded at: https://www.mdpi.com/article/10.3390/foods11010120/s1, Figure S1: Body weight variation during the study. Mice were fed a normal chow (CTR) or a diet with 6.7% (weight/weight) broccoli by-products flour (BF) and were sacrificed after 21 days. Mice were weighed before the start of experiment (1st weighing), after fasting (2nd weighing), 1 week (3rd weighing) and 2 weeks (4th weighing) after the start of experiment, and before euthanasia (5th weighing) at the end of the study. Data are expressed as mean ± SEM (*n* = 5). *** *p <* 0.001, different from CTR for the same time point, according to unpaired Student’s *t*-test, Figure S2: DAPI (4′,6-Diamidino-2-Phenylindole) stained comets (nucleoids) from mice fed with a normal chow (CTR), non-exposed (A) or exposed (B) to formamidopyrimidine DNA glycosylase (FPG), and mice fed with 6.7% (weight/weight) broccoli by-products flour (BF), non-exposed (C) or exposed (D) to FPG. All mice were sacrificed after 21 days of exposure. Comets were isolated from mice leucocytes and visualized in a fluorescence microscope (Olympus BX41 microscope; 400× magnification), Figure S3: Microscopic images (staining with hematoxylin and eosin) of liver and kidney sections from the different groups under study. (A) liver displaying normal architecture from a mouse fed with 6.7% (weight/weight) broccoli by-products flour (BF); (B) cellular tumefaction and focal hydropic degeneration visible at zone 3 in a different mouse fed with BF; (C) multifocal inflammatory infiltrate (lymphocytes and macrophages) in an animal from the CTR group; (D) interstitial chronic nephritis (lymphocytic infiltrate) from a CTR animal, Table S1: Chromatographic and electrospray ionization conditions, Table S2: Chromatographic and electrospray ionization conditions, Table S3: Glucosinolate contents (mmol kg^−1^ DW) in broccoli plant parts (inflorescences, stems and leaves); Supplementary Materials S1: Quantification of glucosinolates in broccoli plant parts (leaves, inflorescences and stems) [65,66,67,68] (References [65,66,67,68] are cited in the Supplementary Materials).
