# Supplementary material for: Effect of a Sub-Chronic Oral Exposure of Broccoli (Brassica oleracea L. Var. Italica) By-Products Flour on the Physiological Parameters of FVB/N Mice: A Pilot Study
Source: Foods. 2022 Jan 4;11(1):120. doi: 10.3390/foods11010120 (PMC8750293; doi:10.3390/foods11010120)
Supplement: Supplementary file 1 [file foods-11-00120-s001.zip › Supplementary Figure S1.pdf]

Supplementary Figure S1

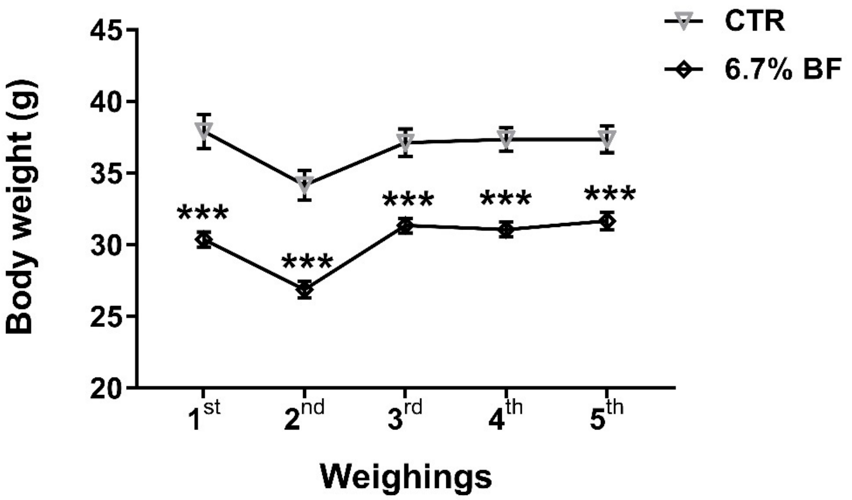

Supplementary Figure S1. Body weight variation during the study. Mice were fed a normal chow (CTR) or a diet with 6.7% (weight/weight) broccoli by-products flour (BF) and were sacrificed after 21 days. Mice were weighed before the start of experiment (1st weighing), after fasting (2nd weighing), 1 week (3rd weighing) and 2 weeks (4th weighing) after the start of experiment, and before euthanasia (5th weighing) at the end of the study. Data are expressed as mean ± SEM (n=5). ****p* < 0.001, different from CTR for the same time point, according to Unpaired Student's t-test.
