# Supplementary material for: Effect of a Sub-Chronic Oral Exposure of Broccoli (Brassica oleracea L. Var. Italica) By-Products Flour on the Physiological Parameters of FVB/N Mice: A Pilot Study
Source: Foods. 2022 Jan 4;11(1):120. doi: 10.3390/foods11010120 (PMC8750293; doi:10.3390/foods11010120)
Supplement: Supplementary file 1 [file foods-11-00120-s001.zip › Supplementary Figure S2.pdf]

Supplementary Figure S2

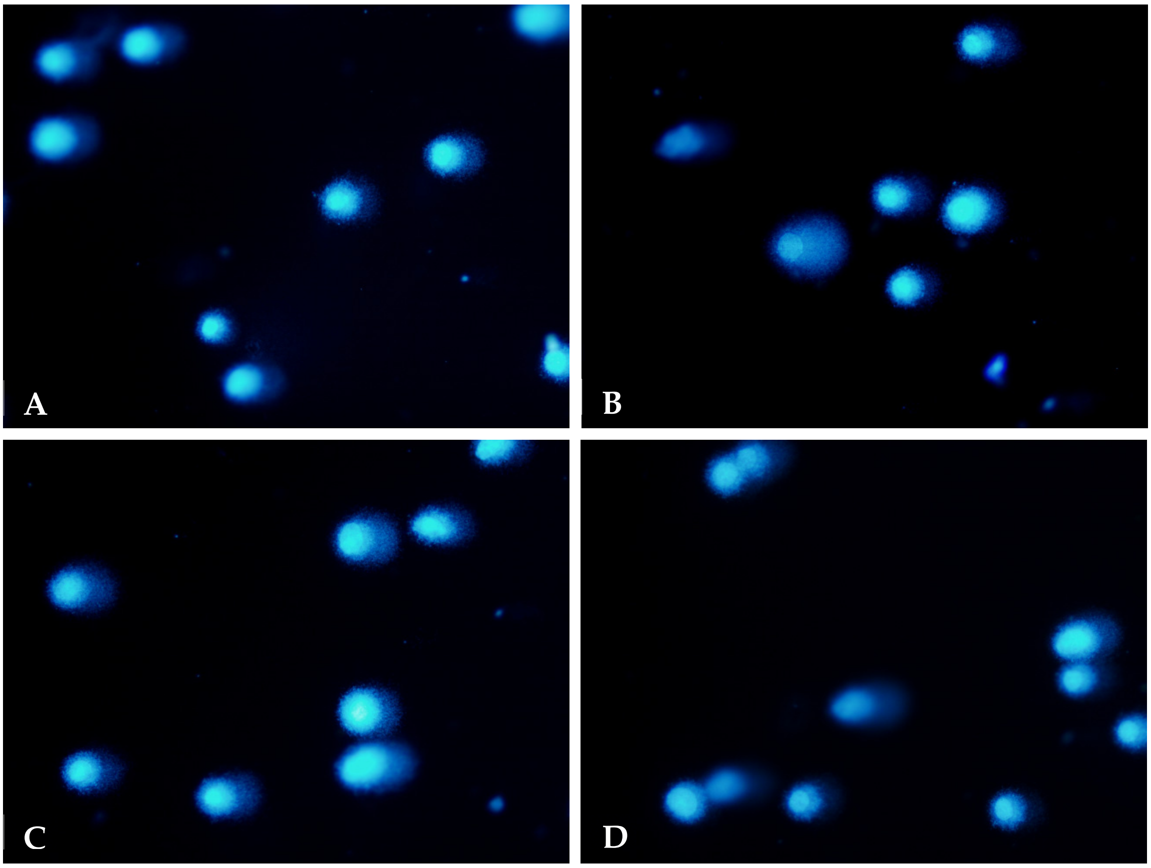

Supplementary Figure S2. DAPI (4',6-Diamidino-2-Phenylindole) stained comets (nucleoids) from mice fed with a normal chow (CTR), non-exposed **(A)** or exposed **(B)** to formamidopyrimidine DNA glycosylase (FPG), and mice fed with 6.7% (weight/weight) broccoli by-products flour (BF), non-exposed **(C)** or exposed **(D)** to FPG. All mice were sacrificed after 21 days of exposure. Comets were isolated from mice leucocytes and visualized in a fluorescence microscope (Olympus BX41 microscope; 400x magnification).
