# Supplementary material for: Effect of a Sub-Chronic Oral Exposure of Broccoli (Brassica oleracea L. Var. Italica) By-Products Flour on the Physiological Parameters of FVB/N Mice: A Pilot Study
Source: Foods. 2022 Jan 4;11(1):120. doi: 10.3390/foods11010120 (PMC8750293; doi:10.3390/foods11010120)
Supplement: Supplementary file 1 [file foods-11-00120-s001.zip › Supplementary Figure S3.pdf]

Supplementary Figure S3

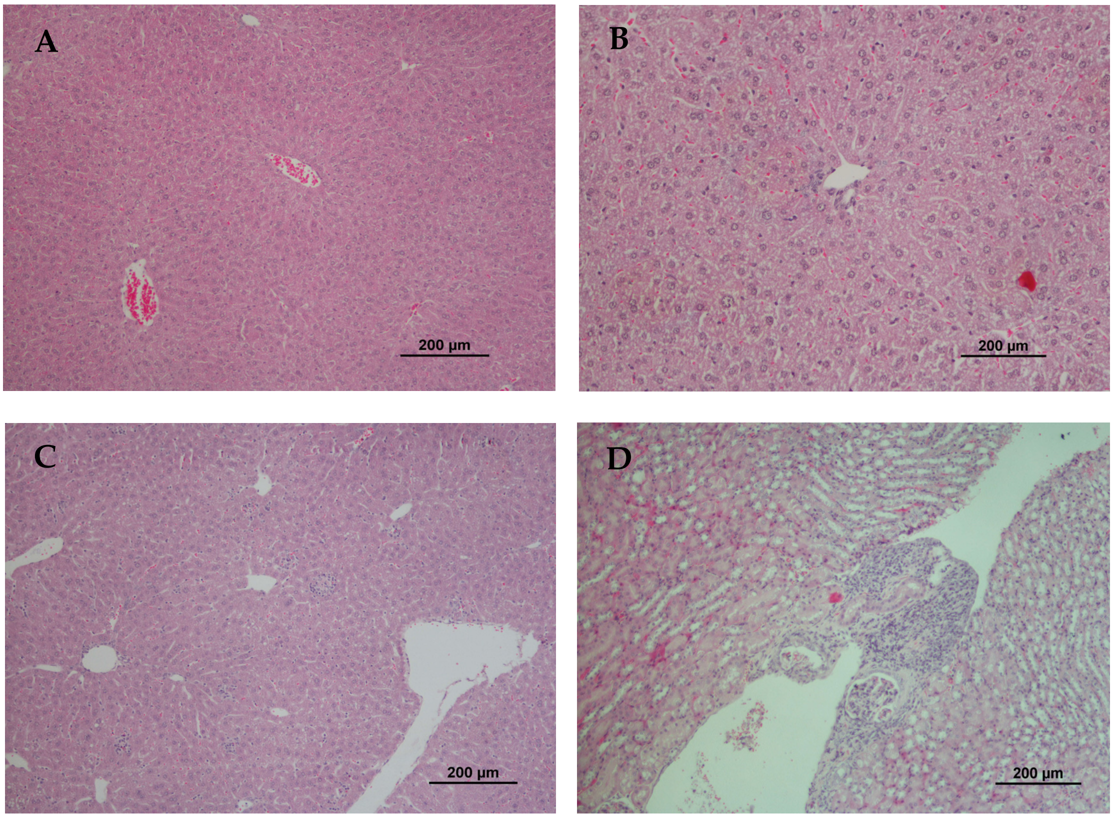

Supplementary Figure S3. Microscopic images (staining with hematoxylin and eosin) of liver and kidney sections from the different groups under study. **A)** liver displaying normal architecture from a mouse fed with 6.7% (weight/weight) broccoli by-products flour (BF); **B)** cellular tumefaction and focal hydropic degeneration visible at zone 3 in a different mouse fed with BF; **C)** multifocal inflammatory infiltrate (lymphocytes and macrophages) in an animal from the CTR group; **D)** interstitial chronic nephritis (lymphocytic infiltrate) from a CTR animal.
