# Supplementary material for: Effect of a Sub-Chronic Oral Exposure of Broccoli (Brassica oleracea L. Var. Italica) By-Products Flour on the Physiological Parameters of FVB/N Mice: A Pilot Study
Source: Foods. 2022 Jan 4;11(1):120. doi: 10.3390/foods11010120 (PMC8750293; doi:10.3390/foods11010120)
Supplement: Supplementary file 1 [file foods-11-00120-s001.zip › Supplementary methods S1.pdf]

Supplementary methods S1

Quantification of glucosinolates in broccoli plant parts (leaves, inflorescences and stems)

The plant samples were analysed for glucosinolates according to a method reported by other authors for the analysis of intact glucosinolates (Francisco et al., 2009; Velasco et al., 2011; Baenas et al., 2012) [65–67], with slight modifications. Briefly, 50 mg of dried sample were accurately weighted and extracted with 1.5 mL of 70% methanol in a thermostated dry bath at 70 °C during 30 min, with vortex mixing each 5 min to facilitate the extraction of the compounds. The extracts were centrifuged at 13.000× g during 15 min (kept at 4 °C). Then, 1 mL of the supernatant was removed, placed in a new tube and evaporated to dryness using a gentle nitrogen stream, at 40 °C. The dried extracts were re-dissolved in 1 mL of ultrapure water (by applying 1min of ultrassonication) and filtered through a 0.2 μm OlimPeak® regenerated cellulose filter (Teknokroma, Barcelona, Spain). Then, 20 μL were injected into the chromatographic system.
The analysis was performed on a Thermo Finnigan Surveyor HPLC system, composed of a quaternary pump, autosampler and PDA detector (Thermo Fisher Scientific, Inc., Waltham, USA). Separation was carried out on an ACE UltraCore 5 SuperC18 column (250 x 4.6 mm, i.d.) (Advanced Chromatography Technologies Ltd, Aberdeen, Scotland), using a mixture of 0.1% trifluoroacetic acid (TFA) in water (phase A) and 0.1% TFA in acetonitrile (phase B) at a flow rate of 1 mL/min. The chromatographic run was performed in gradient mode, starting with 0% B from 0-5 min, reaching 17% at 15 min and maintained until 17 min, increasing to 25% B at 22 min, 35% B at 30 min, 50% B at 35 min and 99% B at 36 min. At 40 min the initial conditions were reset to 0% B in 1 min, and the column was allowed to re-equilibrate for another 10 min until the next injection. The total chromatographic run per injection was 51 min. The glucosinolates were tentatively identified according to the UV-spectra and data available in the bibliography on retention time and elution order (Francisco et al., 2009; Velasco et al., 2011; Baenas et al., 2012; Lee et al., 2013) [65–68]. The chromatograms were recorded at 227 nm, and the compounds were quantified using sinigrin (sinigrin hydrate, from Sigma-Aldrich, St. Louis, MO, USA). The calibration curve was prepared in water in the range of 3.75 μM – 900 μM of sinigrin, the average resulting regression equation was $y = 1.66 \times 10^7 x$ ($R^2 > 0.99$). The 3.75 μM was assumed as the limit of quantification for each compound. The results of glucosinolates quantification were expressed in mmol kg^{-1} of dry weight. For the glucosinolate contents in broccoli plant parts, the one-way ANOVA was performed followed by Tukey's multiple comparison test. Data showed as Mean ± SD. Differences were considered significant when $p < 0.05$.
