# Supplementary material for: Effect of a Sub-Chronic Oral Exposure of Broccoli (Brassica oleracea L. Var. Italica) By-Products Flour on the Physiological Parameters of FVB/N Mice: A Pilot Study
Source: Foods. 2022 Jan 4;11(1):120. doi: 10.3390/foods11010120 (PMC8750293; doi:10.3390/foods11010120)
Supplement: Supplementary file 1 [file foods-11-00120-s001.zip › Supplementary Table S1.pdf]

Supplementary Table S1

Supplementary Table S1. Chromatographic and electrospray ionization conditions.

**Chromatographic conditions**	
**Flow**	0.300
**Injection volume**	10 µL
**Solvent A**	13 mM ammonium acetate pH 7
**Solvent B**	Acetonitrile/formic acid (99.99:0.01, v/v)
**Gradient**	
Time (min.)	%B
0.20	12.00
0.21	70.00
1.00	30.00
1.01	52.00
2.50	52.00
4.00	100.00
7.00	100.00
80.00	12.00

**Electrospray source conditions**	
**Gas temperature (ºC)**	225
**Gas Flow (L/min)**	8
**Nebulizer (psi)**	30
**Seath Gas Heater (ºC)**	350
**Seath Gas Flow (L)**	10
**Capilary (V)**	3500
**VCharging (eV)**	1250
