# Supplementary material for: Effect of a Sub-Chronic Oral Exposure of Broccoli (Brassica oleracea L. Var. Italica) By-Products Flour on the Physiological Parameters of FVB/N Mice: A Pilot Study
Source: Foods. 2022 Jan 4;11(1):120. doi: 10.3390/foods11010120 (PMC8750293; doi:10.3390/foods11010120)
Supplement: Supplementary file 1 [file foods-11-00120-s001.zip › Supplementary Table S2.pdf]

Supplementary Table S2

Supplementary Table S2. Chromatographic and electrospray ionization conditions.

Compound [z]	Rt (min)	Parent ion (*m/z* MS $[M-H]^-$ / m/z MS $[M+H]^+$)	Product ion (*m/z* MS2 $[M-H]^-$ / m/z MS2 $[M+H]^+$)	Dwell	F (eV)	CE (eV)	Cell Acc	Polarity
SFN-NAC	1.108	- / 341.0	- / 178.0	20	80	0	7	Positive
SFN-CYS	0.702	- / 299.0	- / 178.0	20	80	0	7	Positive
DIM	1.66	- / 247.1	- / 105.0	20	70	25	7	Positive
Erucin	0.444	141.1 / -	59.0 / -	20	70	6	7	Negative
SFN	1.644	- / 178.0	- / 114.0	20	75	4	7	Positive
Iberin	1.542	- / 164.0	- / 105.0	20	90	6	7	Positive
I3C	1.284	- / 148.1	- / 77.1	20	70	25	7	Positive
SFN-GSH	1.174	- / 485.0	- / 178.0	20	115	0	7	Positive
GB	1.27	447.2 / -	97 / -	20	80	20	7	Negative
GR	0.626	438.0 / -	196.0 / -	20	90	4	7	Negative
GI	0.648	421.9 / -	357.7 / -	20	100	0	7	Negative
GE	1.211	420.0 / -	97.1 / -	20	60	20	7	Negative

[z] SFN-NAC, Sulforaphane-*N*-acetyl-cysteine; SFN-CYS, Sulforaphane-cysteine; DIM, 3,3'-Diindolylmethane; SFN, Sulforaphane; I3C, indole-3-carbinol; SFN-GSH, Sulforaphane-glutathione; GB, Glucobrassicin; GR, Glucoraphanin; GI, Glucoiberin; GE, Glucoerucin; F, fragmentation energy; CE, collision energy.
