# Supplementary material for: Effect of a Sub-Chronic Oral Exposure of Broccoli (Brassica oleracea L. Var. Italica) By-Products Flour on the Physiological Parameters of FVB/N Mice: A Pilot Study
Source: Foods. 2022 Jan 4;11(1):120. doi: 10.3390/foods11010120 (PMC8750293; doi:10.3390/foods11010120)
Supplement: Supplementary file 1 [file foods-11-00120-s001.zip › Supplementary Table S3.pdf]

Supplementary Table S3

Supplementary Table S3. Glucosinolate contents (mmol kg^{-1} DW) in broccoli plant parts (inflorescences, stems and leaves).

Compounds	Inflorescences	Stems	Leaves
Glucoiberin 3-Methylsulphinylpropyl	0.75 ± 0.01^{A}	1.20 ± 0.09^{B}	0.77 ± 0.02^{A}
Glucoraphanin 4-Methylsulphinylbutyl	11.22 ± 0.25	13.27 ± 1.01	12.23 ± 1.45
**Total aliphatic GLS**	11.97 ± 0.12	14.47 ± 1.12	13.00 ± 1.47
4-Hydroxyglucobrassicin	0.33 ± 0.05^{A}	0.59 ± 0.03^{C}	0.45 ± 0.01^{B}
Glucobrassicin	5.21 ± 0.18^{A}	1.75 ± 0.15^{B}	5.23 ± 0.75^{A}
4-Metoxyglucobrassicin Indol-3-ylmethyl	1.13 ± 0.25	1.02 ± 0.03	1.11 ± 0.04
Neoglucobrassicin 1-Methoxyindol-3-ylmethyl	2.72 ± 0.41^{A}	0.54 ± 0.09^{B}	2.77 ± 1.10^{A}
**Total indolyl GLS**	9.39 ± 0.13	3.90 ± 0.65	9.56 ± 1.27
**Total Glucosinolates**	21.36 ± 0.32	18.37 ± 0.89	22.56 ± 0.44

Data showed as Mean (n=3) ± SD. For each compound, different superscript uppercase letters indicate significant differences (p<0.05) between inflorescences, stems and leaves, according to Tukey's test. DW, dry weight; GLS, glucosinolates.
